# First molecular detection of *Babesia caballi* in camel associated ticks in Uzbekistan, with no evidence of *Anaplasma* or *Rickettsia*

**DOI:** 10.1007/s11259-026-11122-6

**Published:** 2026-03-13

**Authors:** Alisher Safarov, Noureddine Mechouk, Alireza Sazmand, Angela Monica Ionică, Ansorkhon Maksudov, Baurjan Kunisov, Georgiana Deak

**Affiliations:** 1 State Committee of Veterinary and Livestock Development of the Republic of Uzbekistan, Tashkent, Uzbekistan; 2 Samarkand State University of Veterinary Medicine, Livestock and Biotechnologies, Samarkand, Uzbekistan; 3 Department of Parasitology and Parasitic Diseases, University of Agricultural Sciences and Veterinary Medicine of Cluj-Napoca, Calea Mănăștur 3-5, Cluj-Napoca, 400372 Romania; 4 Department of Pathobiology, Faculty of Veterinary Medicine, Bu-Ali Sina University, Hamedan, Iran; 5 Clinical Hospital of Infectious Diseases of Cluj-Napoca, 23 Iuliu Moldovan, Cluj-Napoca, 400348 Romania; 6 Livestock and Biotechnologies, Tashkent Branches of Samarkand State University of Veterinary Medicine, Tashkent, Uzbekistan; 7 Livestock and Biotechnology, Nukus branch of Samarkand State University of Veterinary Medicine, Nukus, Karakalpakstan Uzbekistan

**Keywords:** Asia, Camel, Dromedary, *Babesiacaballi*, Ticks

## Abstract

Dromedary camels (*Camelus dromedarius*) have an important role in food and textile production in arid and semi-arid regions of the world, yet data on their tick-borne pathogens (TBPs) is scarce, especially in Central Asia. This study aimed to: (i) identify the ticks infesting camels from different regions of Uzbekistan, (ii) molecularly detect TBPs in the collected ticks for the first time. In 2024, 290 camels from three regions (Karakalpakstan, Jizzakh, and Navoi) were examined for tick infestation. Ticks were collected, morphologically identified, pooled, and screened using PCR and sequencing for *Babesia*., *Theileria*., *Anaplasma*., and *Rickettsia*. Overall, 32.6% of camels harbored ticks, with 993 specimens collected, belonging to the genera *Hyalomma*, *Dermacentor*, and *Rhipicephalus*, comprising at least six species. Molecular analysis revealed the presence of *Babesia caballi* DNA in two tick pools, marking the first report of this equine pathogen in *Hyalomma* ticks from camels in Uzbekistan. No evidence of other piroplasm, *Anaplasma* or *Rickettsia*. was detected. This study presents an expansion of the known geographic and host range of *B. caballi* and suggests considering camels in TBP surveillance. Further studies combining molecular and serological approaches in multiple host species are needed to elucidate the epidemiological significance of these findings in Central Asia.

**Supplementary Information:**

The online version contains supplementary material available at 10.1007/s11259-026-11122-6.

## Introduction

Dromedary camels (*Camelus dromedarius*), with a world population of more than 41 million, are a valuable source of food, wool, skin, and labor in 47 countries around the world; however, there is little in-depth information about the parasites infecting them compared to other livestock (FAO, 2025). In particular, tick-borne pathogens that pose a serious threat to humans, domestic and wildlife animals, and have not been the focus of molecular studies until recently (Sazmand et al. 2019; Alanazi et al. 2020; Selmi et al., 2019).

Camels can be frequently infested by Ixodidae ticks, especially species of the genera *Hyalomma*, *Dermacentor*, *Amblyomma*, and *Rhipicephalus*, which are widely distributed in arid and semi-arid ecosystems (Ballweber 2009; Getange et al. 2021). Among these, *Hyalomma* spp. are considered the most frequent ticks in camels worldwide and are recognized as vectors of various pathogens affecting both animals and humans (El-Alfy et al. 2024; Soliman et al. 2024). Camels often share grazing areas with equines, cattle, sheep, and goats, creating ecological conditions favorable for tick exchange and interspecific transmission of some pathogens. *Babesia*, *Theileria*, *Anaplasma*, and *Rickettsia* are tick-borne pathogens with global distribution and infect blood cells of mammalian species, some being zoonotic (Uilenberg 1995; Young et al. 2019). Infections with these parasites lead to anemia, loss of productivity, and reduced production of milk and meat (Selmi et al. 2019). Several *Theileria*, *Babesia*, *Anaplasma*, and *Rickettsia* spp. are known to infect camels (Sazmand et al. 2019; Sazmand and Joachim, 2017; Alanazi et al. 2020; Selmi et al., 2019).

In dromedary camels from Egypt, molecular investigations demonstrated the presence of several piroplasmid species, including *T. equi*, *B. caballi*, *B. bigemina*, *B. bovis*, *B. vulpes*, as well as undetermined *Babesia* and *Theileria* spp., using molecular tools (Mahdy et al. 2023).This study provided the first molecular evidence of *B. vulpes* infection in camels, expanding the known host range of this piroplasmid species (Mahdy et al. 2023).

Molecular detection of *Anaplasma* spp. has been reported from camels in several countries in Africa, the Middle East, and Asia, with lower prevalence values than those reported in cattle (El-Alfy et al. 2024). Camels often exhibit mild or no clinical signs, raising the possibility that they act as subclinical carriers (Hekmatimoghaddam et al. 2012; Qablan et al. 2012; Sazmand et al., 2019).The genus *Rickettsia* (Rickettsiales: Rickettsiaceae) comprises obligate intracellular bacteria of major zoonotic concern that are transmitted by ticks through transstadial and, in some species, transovarial routes, enabling ticks to serve both as vectors and reservoirs (Kim 2022). Several *Rickettsia* spp. have been detected in ticks infesting camels, especially in *Hyalomma* spp., which are suitable vectors of pathogenic rickettsiae affecting humans and animals (Alanazi et al. 2020). Although camels are not considered primary hosts for *Rickettsia* spp., their role in sustaining infected tick populations and facilitating human exposure in pastoral systems should not be underestimated, mainly in arid environments with a close contact between humans, camels, and ticks.

In Central Asia, Uzbekistan ranks third in the camel population, with 18,400 heads, only after Turkmenistan and Kazakhstan (FAOSTAT, 2025). In the country, camel breeding is well developed in four regions: the Navoi region (44.3%), the Republic of Karakalpakstan (24.1%), the Bukhara region (11.9%), the Kashkadarya region (8.1%). In other regions, 11.6% of the total number of camels can be found. In Uzbekistan, camels are mainly used for milk, meat, and wool production (Safarov et al. 2024; Kunisov et al. 2024b). Although endoparasites of camels have been studied in Uzbekistan (Sultanov et al. 1973, 1975; Saparov 2006; Azimov et al. 2015; Kunisov et al. 2024a, b; Safarov et al. 2024), there is a shortage of information about tick-borne pathogens (TBPs), although Uzbekistan’s tick fauna comprises 35 species from two families: Ixodidae and Argasidae, distributed across nine genera (Rasulov 2007; Mechouk et al. 2025). In the only previous study, Esonboev et al. (2024) reported *Hyalomma scupense* from camels of the Jizzakh region in Uzbekistan. Likewise, there is no information about the infection of camels with TBPs, although theileriosis has been reported from cattle in several regions of Uzbekistan (Kuchkarova 2023). Considering the shortage of information about ticks and TBPs related to camels and the roles played by camels in the epidemiology of TBPs in Uzbekistan, this study aimed to: (i) identify the ticks infesting camels from different regions of the country, (ii) molecularly detect TBPs in the collected ticks.

## Materials and methods

### Sampling procedures

**Fig. 1:**
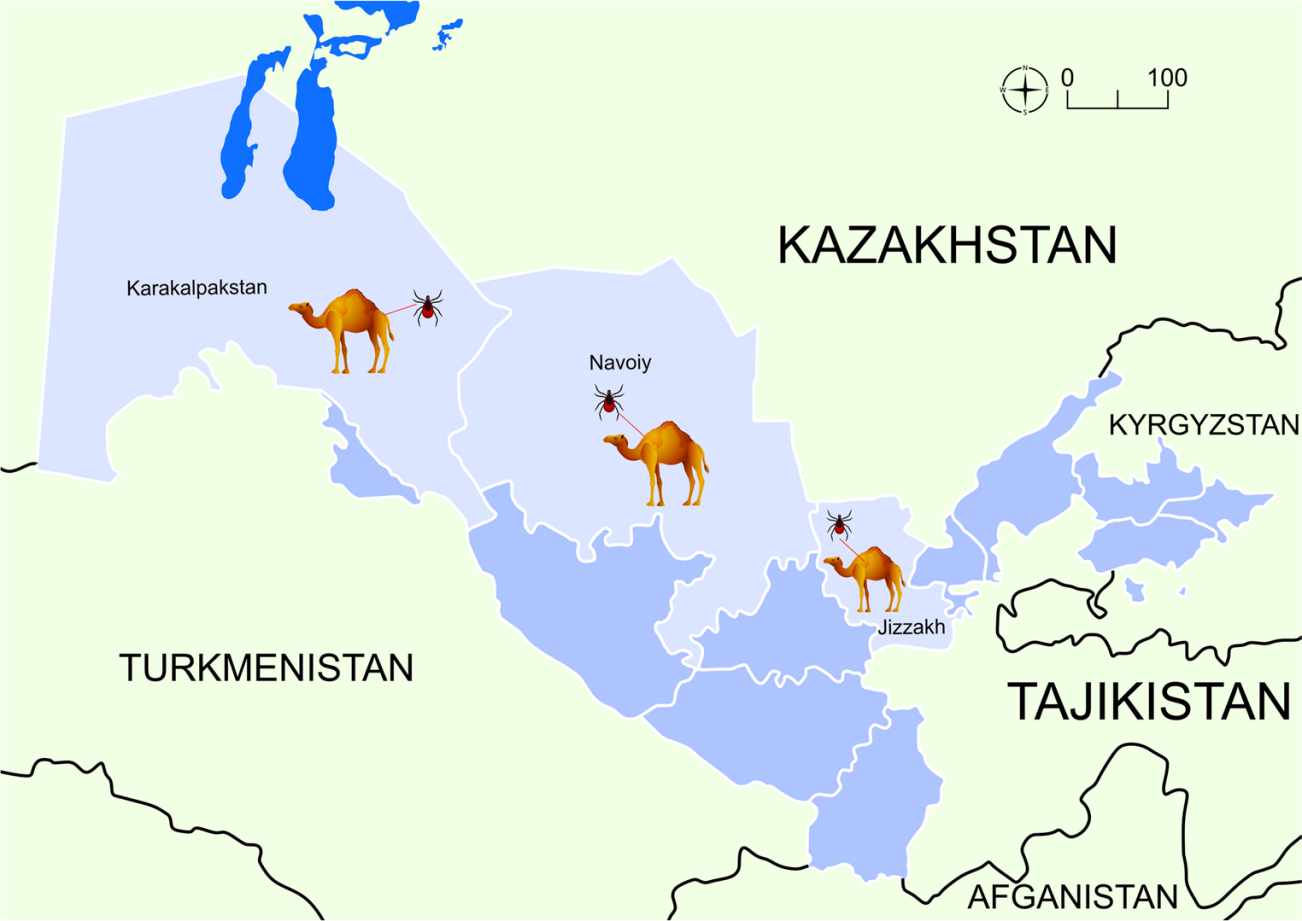
Map showing the investigated areas of Uzbekistan

**Fig. 2:**
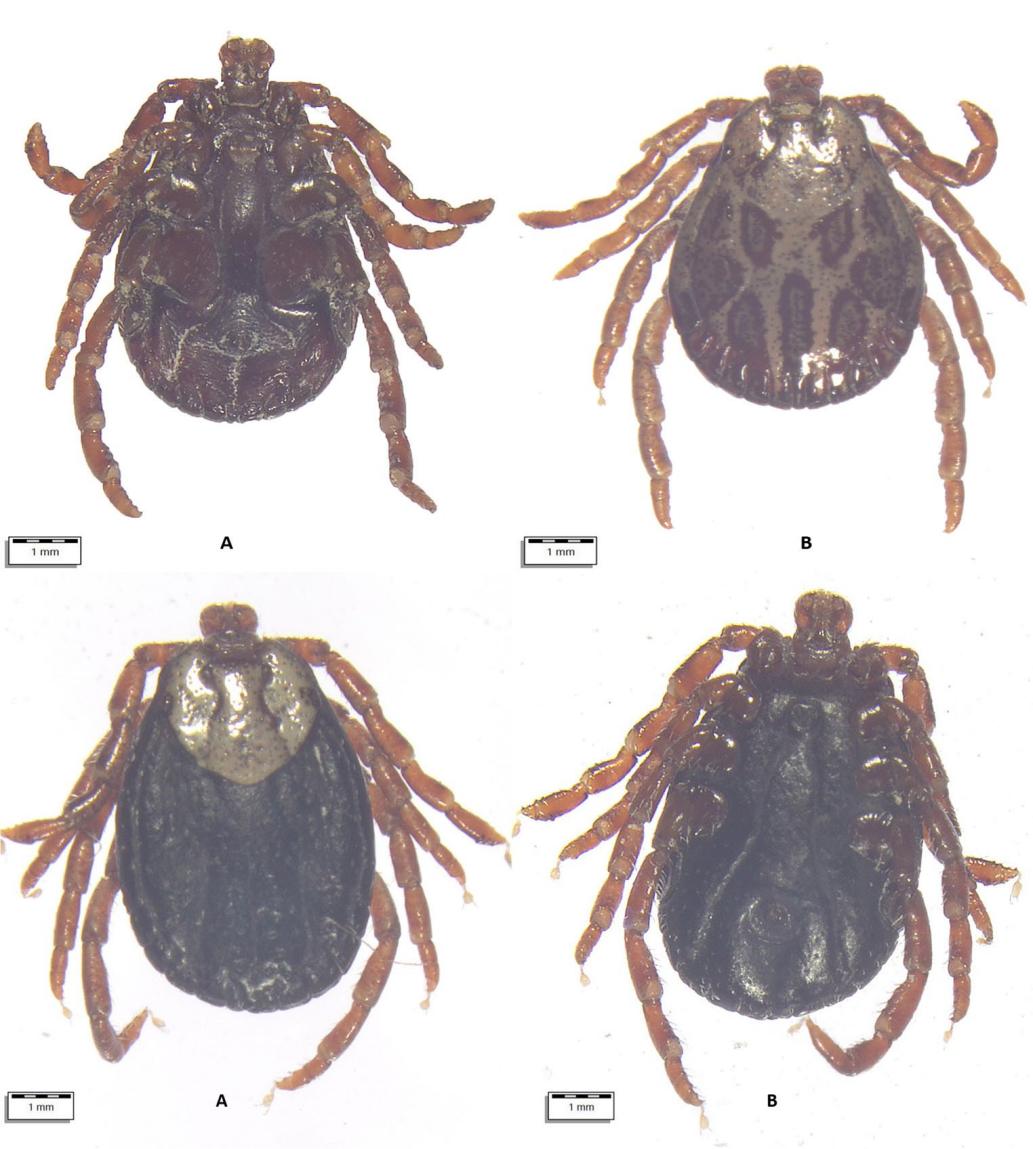
Top: **(A**) *Dermacentor niveus* male ventral view; (**B**) *Dermacentor niveus* male dorsal view. Bottom: **A**. *Dermacentor niveus* female dorsal view. **B**. *Dermacentor niveus* female ventral view

**Fig. 3:**
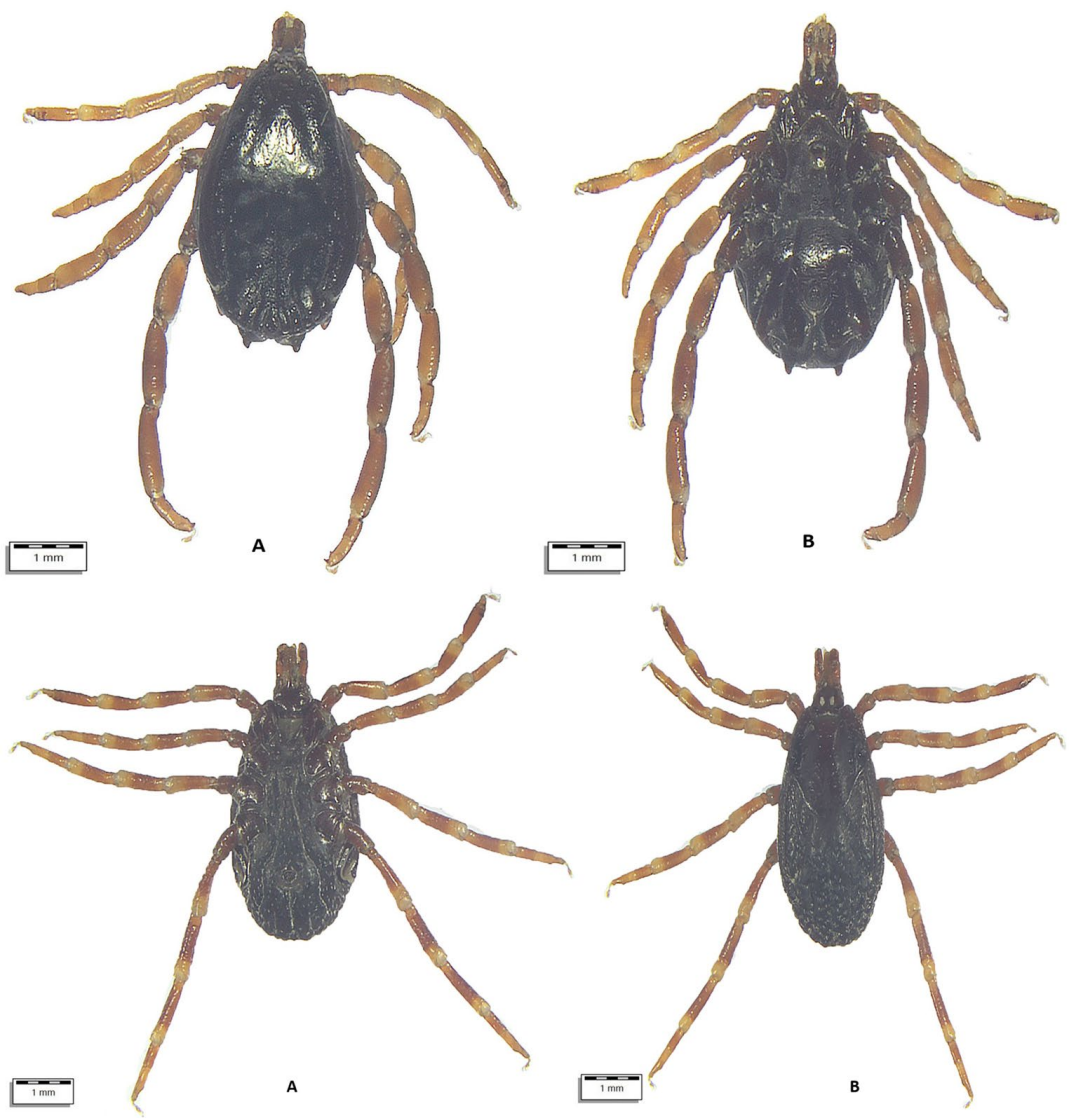
Top (**A**) *Hyalomma anatolicum* male dorsal view, (**B**) *Hyalomma anatolicum* female ventral view. Bottom: **A.** *H. anatolicum* female ventral view, **B**. *H. anatolicum* female dorsal view

**Fig. 4:**
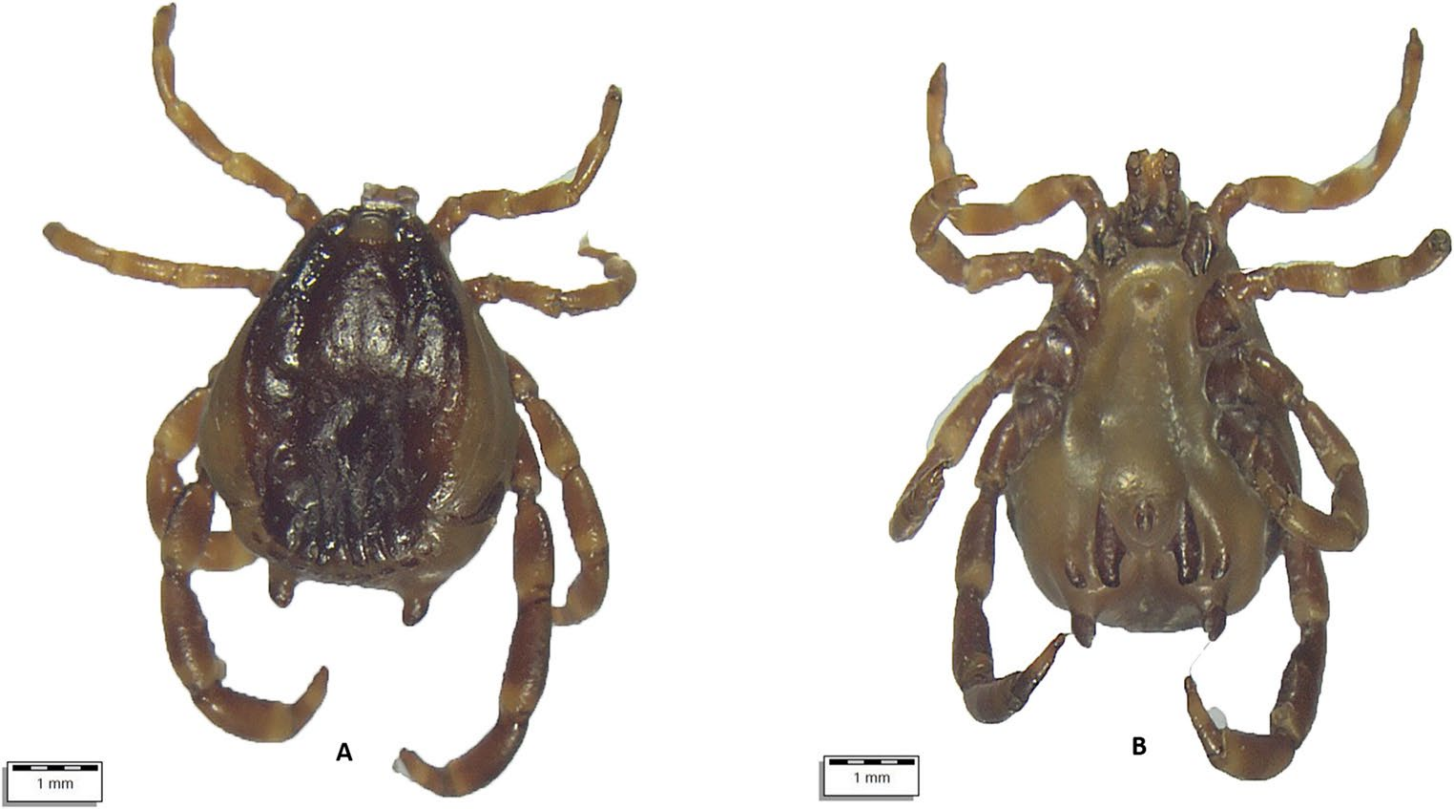
(**A**) *Hyalomma dromedarii* male dorsal view, (**B**) *H. dromedarii* male ventral view

**Fig. 5:**
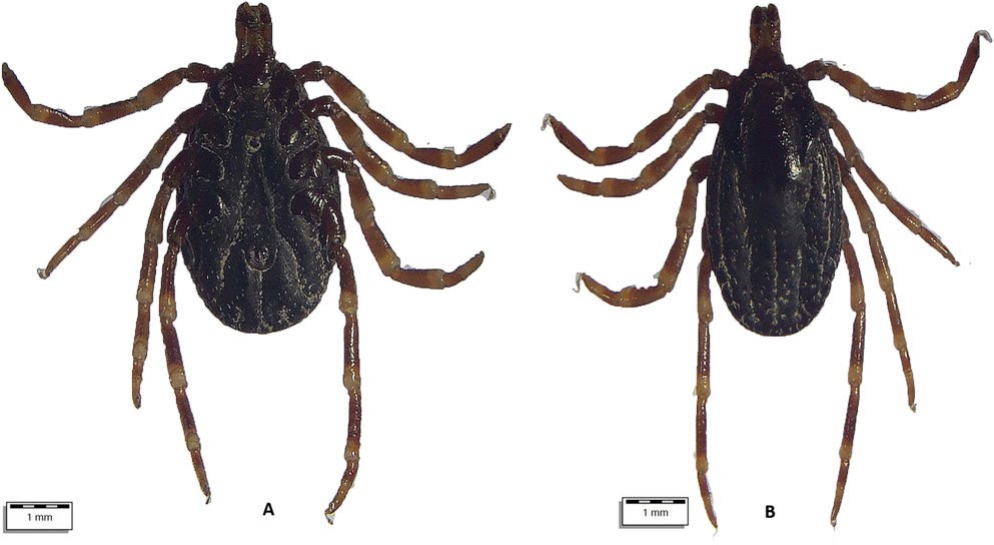
(**A**) *Hyalomma scupense female ventral view*, (**B**) *H. scupens* female dorsal view

**Fig. 6:**
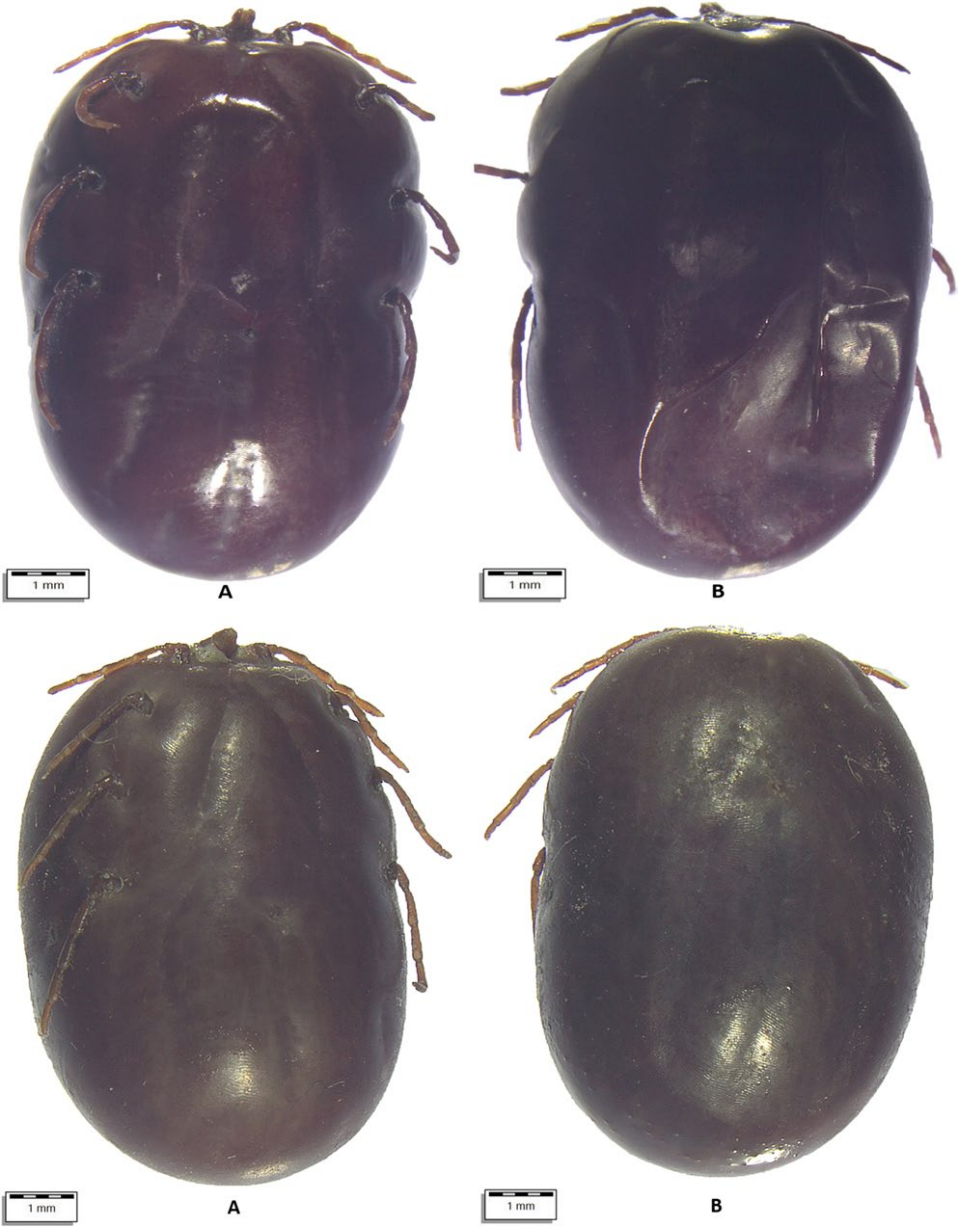
Top: (**A**) *Hyalomma* spp. female ventral view, (**B**) *Hyalomma* spp. female dorsal view. Bottom: (**A**) *Rhipicephalus* spp. female ventral view, (**B**) *Rhipicephalus* spp. dorsal view

From July 10 to August 18, 2024, a total of 290 apparently healthy dromedary camels were inspected for tick infestation. The animals lived in three regions of Uzbekistan with different climatic conditions, i.e., Karakalpakstan (*n* = 122), Jizzakh (*n* = 52), and Navoi (*n* = 116) (Fig. 1). In most cases, camels shared the pasture with horses and cattle. When observed, ticks were collected in individual plastic containers and stored in 70% ethanol. Each tick was individually morphologically identified using valid keys (Estrada-Peña et al. 2018), and photographs were taken using an optical microscope (Olympus BX61) connected to a digital camera (DP72 with Cell∧F imaging software Olympus Corporation, Tokyo, Japan). Figures 2, 3, 4, 5, and 6.

### DNA isolation from camel ticks, molecular analysis by PCR and sequencing

For DNA isolation, ticks were divided into 34 pools, each containing several ticks of the same species from animals from different locations. More specifically, from each geographical region, 4–5 ticks of the same genus were selected and considered an individual pool. When more than one genus/species was detected in one animal from a region, a separate pool was done. Ticks were selected based on their engorgement status, with preference given to less engorged individuals, as these are more likely to yield pathogen DNA with minimal host contamination. In cases where only non-engorged ticks were available, a single tick was selected for analysis (Supplementary file). Each tick was cut in half, and DNA was isolated using a commercial kit (ISOLATE II Genomic DNA Kit, Meridian Bioscience, London, UK), according to the instructions provided by the manufacturer. Specimens were screened by PCR for the presence of DNA of piroplasmids, *Anaplasma* spp., *Ehrlichia* spp., and *Rickettsia* spp. using previously published primers (Table 1) and protocols (Matei et al., 2021; Corduneanu et al. 2023; Mechouk et al., 2024). All PCR reactions included a no-template control consisting of PCR water and a positive control represented by pathogen DNA, previously confirmed by sequencing during other studies. The samples were visualized by electrophoresis (Supplementary file). DNA from individual ticks was not tested when pools were negative. PCR-positive products were purified using the/PCR DNA Fragments Kit (Geneaid Biotech, New Taipei, Taiwan) and bidirectionally sequenced using an external service (Macrogen Europe B.V., Amsterdam, The Netherlands). The sequences were assembled using Geneious software (Biomatters Ltd., New Zealand) and compared to other sequences available in the NCBI GenBank^®^ database by Basic Local Alignment Search Tool (BLAST) analysis.

**Table 1:** Table showing the primers used for the current study

Pathogen	Target gene	Product size (bp)	Forward primer	Reverse primer	Reference
Piroplasmids and *Hepatozoon* spp. (nested PCR)	18 S rRNA	561–620	BTH-1 F: CTGAGAAACGGCTACCACATCT	BTH-1R: TTGCGACCATACTCCCCCCA	Hodžić et al. 2018
			GF2: GTCTTGTAATTGGAATGATGG	GR2: CCAAAGACTTTGATTTCTCTC	Bonnet et al. 2007
SFG *Rickettsia*	*gltA*	381	Rsfg877: GGGGGCCTGCTCACGGCGG	Rsfg1258: ATTGCAAAAAGTACA GTGAACA	Regnery et al. 1991
Anaplasmataceae	16 S rRNA	345	EHR16SF: GGTACCYACAGAAGAAGTCC	EHR16SR: TAGCACTCATCGTTTACAGC	Parola et al. 2000

The phylogenetic analysis was conducted using MEGA X software (Kumar et al., 2018). The sequence attained during the present study was aligned with 42 other *Babesia* spp. and *Theileria equi* sequences, and an additional *Hepatozoon* spp. sequence used as outgroup, downloaded from GenBank^®^, using the MUSCLE algorithm. The evolutionary history was inferred by using the Maximum Likelihood method and Tamura-Nei model (Tamura and Nei 1993), with a discrete Gamma distribution used to model evolutionary rate differences among sites (5 categories (+ *G*, parameter = 0.1581).

### Statistical analysis

Prevalence (i.e., proportion of hosts infected with ticks), tick burden (i.e., arithmetic mean number of ticks per infected host), and pathogen infection rate were estimated using the Quantitative Parasitology program recommended by Rózsa et al. (2000). Confidence intervals (95% CI) were calculated. Differences among regions were assessed by chi-square testing and were considered significant at *p* < 0.005.

## Results

Of the 290 camels examined, 88 (32.59%; 95% CI: 27.04–38.5) were infested by ticks, with a total of 993 specimens collected: 482 (48.54%, 95% CI: 45.4–51.6) males, 439 (44.2%, 95% CI: 41.1–47.3) females and 72 (7.2%, 95% CI: 5.8–9.03) nymphs. Ticks have been recorded on camels distributed across all three study regions: Karakalpakstan – 51 (41.8%; 95% CI: 32.9–51); Jizzakh – 21 (18.1%; 95% CI: 11.6–26.3); Navoiy – 16 (30.7%; 95% CI: 18.7–45.1). The prevalence of tick infestations was significantly higher in Karakalpakstan region when compared to Navoiy (*p* < 0.0001). The differences between Jizzakh and the other two regions were not statistically significant (*p* = 0.18 against Karakalpakstan and *p* = 0.08 against Navoiy). The highest intensity of infestation was recorded in Karakalpakstan, with a median of 11 ticks/camel (IQR: 10–16), followed by Navoiy with a median of 4 ticks/animal (IQR: 3–10), and Jizzakh, with a median of 3 ticks/camel (IQR: 1.5–7.5). The complete data about camels, including the collection date, age, sex, and locality, are shown in the Supplementary file.

All ticks were morphologically identified as belonging to three genera (*Hyalomma*, *Dermacentor*, *Rhipicephalus*) and at least six species (Table 2; Supplementary file). Some ticks were not identified to the species level due to their poor preservation and invisible morphological structures. Information on the age and sex of the affected camels and their origin is presented in the Supplementary file.

**Table 2:** Tick species identified and their development stages

Tick species	Camels affected		Total	Developmental stages		
	*N*/88 (%)	95% CI		Female	Male	Nymph
*Hyalomma anatolicum *(Fig. 3)	72 (81.82)	72.16–89.24	440	53	387	0
*Hyalomma dromedarii *(Fig. 4)	17 (19.32)	11.68–29.12	49	0	49	0
*Hyalomma scupense*(Fig. 5)	1 (1.14)	0.03–6.17	1	1	0	0
*Hyalomma* spp. (Fig. 6)	71 (80.68)	70.88–88.32	386	308	6	72
*Dermacentor niveus *(Fig. 2)	3 (3.41)	0.71–9.64	108	68	40	0
*Rhipicephalus sanguineus* s.l.	7 (7.95)	3.26–15.7	9	9	0	0

**Fig. 7:**
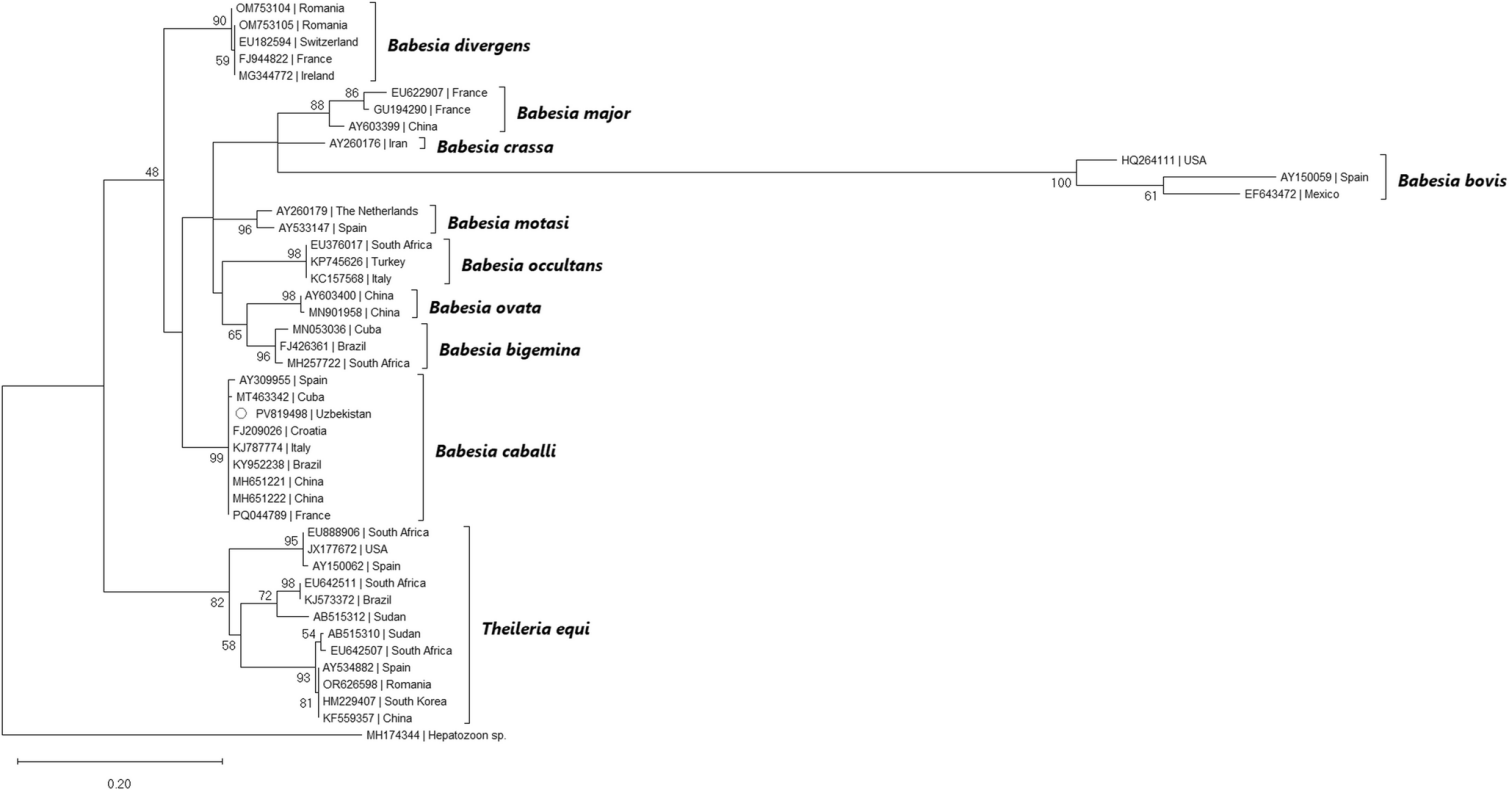
Bootstrap consensus tree. The percentage of trees in which the associated taxa clustered together is shown next to the branches (values below 40% not shown). The tree is drawn to scale, with branch lengths measured in the number of substitutions per site. This analysis involved *Babesia* nucleotide sequences, and one *Hepatozoon* sequence used as outgroup

Out of 34 tested pools (131 ticks), two were positive for piroplasms (Supplementary file). Sequencing revealed *Babesia caballi* in the two positive samples, one from a pool of *H. anatolicum* male ticks from Karakalpakstan and the second from a pool of *D. niveus* from Jizzakh, both showing a 100% similarity with over 30 other entries from GenBank, e.g., in the blood of equines worldwide, in France (PQ044790), Spain (AY534883), Brazil (KY952238), Cuba (MT463341), South Africa (EU642512). The sequence was deposited in GenBank under the accession number PV819498. *Theileria* spp. was not identified in none of the two positive samples for piroplasmids. The phylogenetic analysis also placed the herein obtained sequence in a clade including exclusively *B. caballi* isolates (Fig. 7).

All samples tested negative for Anaplasmataceae and *Rickettsia* spp.

## Discussion

This study reports the first molecular identification of *B. caballi* in Uzbekistan, expanding current knowledge on tick-borne parasites in Central Asia. Of note, the detection of *B. caballi* in ticks collected from camels in Uzbekistan suggests the potential role of camels and their associated ticks in the epidemiology of equine piroplasms in Central Asia. Previously, in neighboring Kazakhstan, *B. caballi* was identified in *Dermacentor marginatus* collected from cattle (Chimkent − 5.6% and Saryagash district − 30%) and horses (10%, Karabulak district). In another study from Kazakhstan, *Babesia caballi* was not detected in *Hyalomma asiaticum* collected from camels and *Rhipicephalus turanicus* ticks collected from dogs, using molecular techniques (Chunli et al. 2021). Additionally, in Kyrgyzstan, *B. caballi* was reported in the blood of 8.4% (160/1900) of imported donkeys from the Osh region, southwestern Kyrgyzstan (Wu et al. 2024).

Although *B. caballi* is normally associated with equines, the identification in ticks from camels could represent an interspecific transmission, which might have occurred in mixed-grazing systems. In many parts of Africa and Asia, including Uzbekistan, camels often share grazing areas with other animals such as cattle and equines, particularly horses and donkeys (Alanazi et al. 2020). This practice of co-grazing could facilitate exposure to ticks, making cross-species transmission of TBPs possible. *Babesia caballi* was identified in the blood of camels using molecular tools in Iraq (39.5%, 31.6%; Hussein et al. 2015), Iran (19.3%; Mirahmadi et al. 2022), Jordan (6%; Qablan et al. 2012), and Egypt (5.4–8.3%; Mahdy et al. 2023; Amer et al. 2024) (Table 3). *Theileria equi*, the causative agent of equine theileriosis, has been identified in the blood of camels in Egypt (41%, 4.4%; Mahdy et al. 2023), Iraq (23.68% Hussein et al. 2015), Jordan (4%; Qablan et al. 2012), and Iran (2.8%; Bahrami et al., 2016). It is known that equine piroplasmosis (EP), caused by *B. caballi* and *T. equi*, causes significant economic losses in the equine industry.

**Table 3:** Table shows a revision of all studies investigating *Babesia caballi* and *Theileria equi* in camels or camel-associated ticks

Pathogen	Host	Prevalence % (positive/total)	Country	Reference
*Babesia caballi*	*Camelus dromedarius*	5.9 (2/34)	Uzbekistan	Current study
	*Camelus dromedarius*	6 (6/100)	Jordan	Qablan et al. 2012
	*Camelus dromedarius*	5.4 (11/203)	Egypt	Mahdy et al. 2023
	*Camelus dromedarius*	1.2 (3/248)	Iran	Bahrami et al. 2017
	*Camelus dromedarius*	39.5 (15/38)	Iraq	Jasim et al. 2015
	*Camelus dromedarius*	83.8 (62/74)	Iraq	Al-Obaidi et al. 2021
	*Camelus dromedarius*	62.2 (51/82)	Egypt	Kuraa and Malek 2024
	*Camelus dromedarius*	8.3 (15/181)	Egypt	Amer et al. 2024
	*Camelus dromedarius*	19.3 (27/140)	Iran	Mirahmadi et al. 2022
	*Hyalomma dromaderii*	0.2 (1/593)	Nigeria	Onyiche et al. 2020
*Theileria equi*	*Camelus dromedarius*	4 (4/100)	Jordan	Qablan et al. 2012
	*Camelus dromedarius*	41 (84/203)	Egypt	Mahdy et al. 2023
	*Camelus dromedarius*	4.4 (8/181)	Egypt	Amer et al., 2024
	*Camelus dromedarius*	2.8 (7/248)	Iran	Bahrami et al. 2017
	*Camelus dromedarius*	23.7 (9/38)	Iraq	Jasim et al. 2015

Economic losses include treatment costs, abortions, loss of activity, and death. Clinical signs of equine babesiosis and theileriosis vary, but share fever, anemia, inappetence, oedema, icterus, hepatomegaly, splenomegaly, and death in some cases (Onyiche et al. 2019). Dromedary camels infected with *B. caballi* and *T. equi*, including camels in this study, however, have been reported to be apparently healthy or have no clinical signs usually associated with piroplasmosis (Qablan et al. 2012). Furthermore, dromedaries mainly free-roam in arid and semi-arid areas where other livestock, including horses, cannot resist (Qablan et al. 2012), supporting the hypothesis that infection of camels with equid piroplasms could circulate in camel populations without frequent involvement of the equine hosts. This hypothesis is further supported by the transovarial and transstadial transmission of *B. caballi* and *T. equi* in Ixodidae ticks (De Waal and Potgieter 1987; De Waal 1990; Scoles and Ueti 2015; Nadal et al. 2022; Ravindran et al., 2023). (Nadal et al. 2022; Ravindran et al. 2023) Hence, the molecular detection of equine piroplasms in ticks from camels suggests inclusion of camels in epidemiological surveillance programs for piroplasmoses, especially in regions like Central Asia.

Prior to this study, in Uzbekistan, some faunistic researches were conducted on ticks infesting camels (Kuklina, 1976; Rasulov 2007). However, no data were provided on how many of these tick species are found on camels. Analysis of literature data shows that ticks of the genus *Hyalomma* are the most common species infesting camels worldwide, many of which can serve as vectors for piroplasms (El Kady 1998; Elghali and Hassan 2009; Fard et al. 2012; Shiri et al. 2024). In Kazakhstan, *B. caballi* was detected in *Dermacentor marginatus* (Chunli et al. 2021), while in the present study, it was identified only in *Hyalomma* sp. It is worth noting that the detection of a pathogen DNA in ticks does not confirm the host’s infection (Krupa et al. 2024). To address this, future studies should consider tick dissection and examination of salivary glands to distinguish between DNA carriage resulting from bloodmeal and active infection, correlated with blood screening of the same animals (Cabezas-Cruz et al. 2018). A possible explanation for the negative molecular results regarding *Anaplasma* and *Rickettsia* spp. could be the inherently low infection rates of these bacteria in camel-associated ticks in the studied regions, combined with the limited number of tested pools. Moreover, the pooling strategy and the use of conventional PCR may have reduced the sensitivity for detecting low-level or mixed infections. It is also possible that the local environmental conditions, such as ecological and climatic, and the predominance of *Hyalomma* spp. more commonly associated with piroplasmids than with *Anaplasma* or *Rickettsia* has contributed to the absence of positive findings. The absence of *Anaplasma* spp., *Theileria* spp., and *Rickettsia* spp. DNA in the tested ticks might either reflect an absence in Uzbekistan or might be due to the sample size and pooling strategy.

Considering the previous reports of *Anaplasma* spp. in camels and ticks in other regions (Selmi et al. 2019; Alanazi et al. 2020), ongoing monitoring remains important.

The present study had several limitations that should be mentioned. First, no pathogen testing was conducted on the blood of the same host animal. Such a thing would have made possible the assessment of pathogen transmission and could have correlated molecular findings in ticks with systemic infections. Secondly, the use of conventional PCR might have led to an underestimation of mixed piroplasmid infections, as its sensitivity is limited compared to more advanced techniques (Criado-Fornelio et al. 2003). Third, the amplification of a single target gene for each of the pathogens may have slightly reduced the reliability of detection results. Additionally, a proportion of collected ticks could only be identified to the genus level due to poor preservation, which might have restricted the precise ecological interpretation and showed imprecise definitive conclusions regarding species-specific vector competence and pathogen–vector associations.

## Conclusion

This study, involving a large sample of 290 investigated camels and nearly 1000 associated ticks, provides the first molecular confirmation of *B. caballi* in *Hyalomma* spp. ticks collected from camels in Uzbekistan are expanding the current knowledge on tick-borne pathogens in Central Asia, and opening doors for further investigations into the epidemiological role of camels in the transmission of *B. caballi*, a pathogen traditionally associated with equines. None if the pooled tick samples were positive for *Anaplasma* or *Ehrlichia*. To elucidate this, parallel screening of equines in the region and comparison of the genetic characteristics of the parasites detected in two hosts are essential. Considering the co-grazing system is common in Central Asia, these results suggest that camels should be included in the regional screening programs for tick-borne pathogens.

## Supplementary Information

Below is the link to the electronic supplementary material.

Supplementary Material 1 (XLSX 2.65 MB)

## Data Availability

All relevant data are enclosed within the manuscript.
